# Anaesthesiologist’s Attitude and Behavior Toward Postoperative Pain Management in Turkey

**DOI:** 10.5152/TJAR.2021.1561

**Published:** 2022-02-01

**Authors:** Emin Tunç Demir, Mesut Erbaş

**Affiliations:** 1 Department of Anaesthesiology, Reanimation and Intensive Care, Aydın Atatürk State Hospital, Aydın, Turkey; 2 Department of Anaesthesiology and Reanimation, Çanakkale Onsekiz Mart University, Çanakkale, Turkey

**Keywords:** Multimodal analgesia, pain assessment, pain team, postoperative pain

## Abstract

**Objective:**

**:** This study aims to evaluate the approach of anaesthesiologist in Turkey and their applications toward postoperative pain treatment and in addition to raise awareness in this regard.

**Methods:**

The target audience of this descriptive survey study was physician members of the Turkish Society of Anaesthesiology and Reanimation, who were volunteering/accepting to participate in the study. The doctors were contacted via their e-mail addresses. Data were collected online, between October 10, 2016, and November 30, 2016, using a web-based (SurveyMonkey®, https://tr.surveymonkey.com/) questionnaire form, and the data were analyzed by the Statistical Package for the Social Sciences (SPSS) version 20 software (IBM Corp.; Armonk, NY, USA). Descriptive data were presented with frequency, percentage, mean, standard deviation, median, minimum, and maximum values.

**Results:**

A total of 315 people were included in the study. Around 34.9% anaesthesiologists had 5-10 years of professional experience and 61.9% of the anaesthesiologists stated that they routinely check the patients’ pain level in the postoperative period. Multimodal analgesia is mostly preferred (25.3%) after major surgical intervention. Around 71.9% of the participants stated that they cannot find the required time for postoperative analgesia in their institution, and they associated this matter with excessive workload and lack of staff time.

**Conclusion:**

In this study, we found that anaesthesiologists in Turkey are doing the follow-up of patients during the postoperative period pain-wise and that they use specific pain scales. Anaesthesiologists think that postoperative pain treatment is not done effectively and time required for the pain treatment is not enough. They also stated that a separate team should be formed for postoperative pain management in the hospital. We believe that this study will raise awareness on this issue and will contribute to the creation of algorithms for postoperative pain treatment, the establishment of pain teams, and the provision of more effective and safer health services.

**Main Points**

- Anaesthesiologist in Turkey are giving postoperative pain treatment support and they are using various pain level scales.
- Anaesthesiologists were aware of the necessity of postoperative pain treatment and took an active role in the unit they work.
- Anaesthesiologists participating in our study also stated that they benefited from national and international protocols, but mostly they used various methods shaped by their personal experiences depending on situational needs.
- Considering the current workload of the anaesthesiologists, it was concluded that a separate pain team, which should be responsible for postoperative pain treatment, should be formed.

## Introduction

Postoperative pain is an acute pain that starts with a surgical incision and gradually decreases with the healing of the tissue.^1^ Acute pain encountered in the postoperative period affects the general well-being of the patients by causing conditions that disrupt the patient comfort such as nausea, vomiting, and dizziness. Also, it increases postoperative morbidity and extends the duration of hospital stay. On the other hand, minimizing postoperative pain is an ethical obligation.^2,3^

Despite the advances in non-invasive and invasive pain management techniques, postoperative pain management is far from optimal.^3^ Studies have shown that 50-70% of patients experience postoperative pain in moderate or severe form and the pain treatment is insufficient.^4,5^ Difficulties in evaluating pain are caused by the fact that the same pain is experienced differently by different patients and that it is affected by many factors such as gender, age, and ethnic background. For that reason, it is not possible to reach a standard in the assessment of pain level among individuals, in addition to that it is not likely for the patient to define their pain levels correctly during the postoperative period. Inadequate treatment of postoperative pain after all these difficulties may contribute to an increase in stress and depression.^6^

The aim of this study is to further investigate the follow-ups and experiences of patients with acute pain and the applied analgesia methods by anaesthesiologist in Turkey.

## Methods

The population of this descriptive study is anaesthesiologists from Turkey who work in various health institutions. The data were obtained using a questionnaire through Surveymonkey^®^ (Copyright 1999-2020 SurveyMonkey, https://tr.surveymonkey.com). The link for the survey was posted to all email addresses registered in the Turkish Society of Anaesthesiology and Reanimation member database (2800 members). A second notification was sent to the same email addresses on October 2016 with a notice of not to reply to the survey if it was replied after the first email. Participation in the survey was voluntary and anonymous, and there was no compensation for participation. 

The questionnaire, which consisted of 30 questions, included questions about sociodemographic characteristics, workplace characteristics, the way physicians manage postoperative pain treatment, and their approach toward patient follow-up.

Ethics committee approval was received for this study from the ethics committee of Çanakkale Onsekiz Mart University (Date: 7 June 2017; Approval number: 2017.11).

### Statistical Analysis

Statistical Package for the Social Sciences software (IBM SPSS Statistics for Windows, Version 20.0. IBM Corp., Armonk, NY, USA) package program was used in the statistical analysis of the data, the results were shown as mean ± standard deviation, median (the smallest value − the largest value), and percentages of variables.

## Results

**Figure 1.**
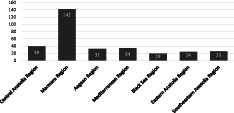
Distribution of anaesthesiologists by geographical regions.

Of the 2800 mail recipients, all anaesthesiologists working in the hospitals, 315 completed the survey form, making the response rate to be 11.25%. A total of 315 people, 62.2% (n = 196) female and 37.8% (n = 119) male, participated in the study. Around 34.9% of the anaesthesiologists participating in the study had 5-10 years of professional experience and 40% of the participants were physicians working in a state hospital. The demographic characteristics of the anaesthesiologists participating in the study are shown in Table 1. Anaesthesiologists from 46 cities (there are 7 regions and 81 cities in Turkey) participated in the study. Among the answers received, respondents from big cities had a highlighted importance in the study (Figure 1). Around 61.9% of the anaesthesiologists participating in the questionnaire stated that they routinely performed the pain controls of the patients in the postoperative period and the visual analog scale was the scale they used the most (41.3%). The approaches of the anaesthesiologists participating in the questionnaire are shown in Table 2. Participants stated that their most important goal in reducing pain in the postoperative period was patient satisfaction (33.6%). In addition, 71.9% of the participants stated that postoperative analgesia treatment duration was not enough in their institution (Table 3). Anaesthesiologists stated that they mostly preferred multimodal analgesia (25.3%) after major surgical intervention in the postoperative period, and their opinions and practical applications on this issue are shown in Table 4 and Table 5, respectively. Participants’ opinions about preemptive analgesia applications and non-pharmacological methods are shown in Table 6.

## Discussion

Our study showed us that anaesthesiologists in Turkey are giving postoperative pain treatment support and that they are using various pain level scales. It was observed that intermittent non-steroidal anti-inflammatory drug (NSAID) administration and opioids were preferred for postoperative pain treatment of the patients. On the other hand, anaesthesiologists think that there should be a separate team for postoperative pain and stated that they use methods based on their own experience instead of routine protocols.

Özer and Bölükbaşı^7^ reported that 93.7% of the patients who underwent surgery complained of severe pain in the postoperative period. Since pain is an important postoperative problem, many guidelines on postoperative pain management are published around the world. Anaesthesiologists participating in our study also stated that they benefited from national and international protocols, but mostly they used various methods shaped by their personal experiences depending on conditional needs.

Although the pain in the postoperative period is acute, it can become chronic when not managed. According to Macrae’s^8^ study, it was reported that the post-surgical chronic pain of patients who underwent surgical procedures in the United Kingdom and the United States varied depending on the type of surgery and the ratio ranged between 5% and 85%. In our study, anaesthesiologists stated that their most important aim was to increase patient satisfaction. Other goals regarding postoperative pain were observed to be reducing the side effects of insufficient pain management and reducing postoperative morbidity.

In multimodal analgesia, there are local anaesthetic, NSAID, opioid, paracetamol drugs as well as anxiolytic and neuroleptic, anticonvulsant, antidepressant drugs called adjuvant analgesics. In addition to pharmacological treatment, non-pharmacological treatments are also applied.^9,10^ In the study conducted by Lorentzen et al.^11^ with 424 patients, strong opioids, weak opioids, NSAIDs, and paracetamol group drugs were administered to the patients alone or in combination. The interview with the patients showed that 4.2% of the patients did not use analgesics, and the pain control was not sufficient in 45.5% of the patients, even though 88.4% of the patients were satisfied or very satisfied with the pain treatment.^11^ A significant portion of the participants stated that they preferred multimodal analgesia, especially after major surgical interventions. In addition, only 6% of the anaesthesiologists participating in the study stated that they used non-pharmacological methods for postoperative pain treatment. As a non-pharmacological method, psychological premedication is mostly preferred. 

Opioids administered intravenous (IV) with patient-controlled analgesia (PCA) are more effective than conventional intramuscular (IM) or IV opioid applications. In the literature, it has been shown that the risk of respiratory depression in PCA applications (0.25-0.50%) is lower than intermittent IM opioid administration (0.9%) and it also does not affect respiratory functions.^12,13^ In the “Postoperative Pain Management Guide” published by the Turkish Society of Anaesthesiology and Reanimation, it is reported that PCA is superior to IM injection applications, provides effective analgesia, and causes less side effects.^14^

In addition, the participants stated that they cannot adequately manage the postoperative pain in the institution where they are working, and this situation is mostly associated with the excess workload and lack of personnel/time. They also stated that they think a separate team should be formed for postoperative pain management in the hospital.

It is quite clear that postoperative pain as a manifestation of acute pain has more complex pathophysiological effects than usually thought. It is important to understand that postoperative pain management is not only a humanitarian task to ease the suffering of patient and increase patient satisfaction but also that acute postoperative pain management is associated with postoperative morbidity and mortality. As a result of our study, it was determined that anaesthesiologists in our country were aware of the necessity of postoperative pain treatment and took an active role in the unit they work. 

## Conclusion

Considering the current workload of the anaesthesiologists, it was concluded that a separate pain team, which should be responsible for postoperative pain treatment, should be formed. That way, we think that it will be possible to use different analgesia methods and that patient follow-up in the postoperative period will be performed more efficiently with a holistic and multidisciplinary approach. 

## Figures and Tables

**Table 1.** Anaesthesiologist’s Demographic Statistics

Variables	n (%)
**Gender**	
Female	196 (62.2)
Male	119 (37.8)
**Institutions of work**	
Public hospital	126 (40.0)
University hospital	86 (27.3)
Education and research hospital	68 (21.6)
Private hospital	35 (11.1)
**Working years**	
0-3 years	59 (18.7)
3-5 years	52 (16.5)
5-10 years	110 (34.9)
10-20 years	66 (21.0)
Over 20 years	28 (8.9)

%: column percentage

**Table 2.** Distribution of Participants According to Their Approach Toward Postoperative Pain Treatment

Variables	n (%)
**Do you routinely evaluate your patients’ postoperative pain level status?**	
Yes	195 (61.9)
No	43 (13.7)
Sometimes	77 (24.4)
**Do you use measurement methods and scales to detect pain levels in postoperative patients?**	
Yes	178 (56.9)
No	135 (43.1)
If you are using measurement methods and pain scales for pain level detection, what scales do you use frequently?*	
VAS	177 (41.3)
Numerical Rating Scales	54 (12.6)
The Faces Pain Scale	77 (17.9)
The McGill Pain Questionnaire	3 (0.7)
The West Haven-Yale Multidimensional Pain Inventory	-
The Dartmouth Pain Questionnaire	-
I evaluate according to the patient’s reaction to pain	118 (27.5)
**How often do you use the postoperative pain monitoring measurement methods and scales?**	
1 time a day	43 (14.0)
2 times a day	42 (13.6)
More than 2 times a day	48 (15.6)
When needed	175 (56.8)
**Is there a “pain team” serving postoperative patients in the hospital where you work?**	
Yes	46 (14.7)
No	267 (85.3)
Who works in pain teams in institutions with pain teams?*	
Anaesthesiologist	29 (29.3)
Anaesthesia assistant	25 (25.3)
Pain technician	24 (24.2)
Pain nurse	19 (19.2)
Surgeon	2 (2.0)
Physiotherapist	-

%: column percentage, %*: calculated over the total answer given, VAS: Visual Analogue Scale

**Table 3.** Approach to Pain Management at the Institution of Employment

Variables	n (%)
Do you use protocols during postoperative pain treatment? *	
Yes, international protocols	52 (16.4)
Yes, national protocols	29 (9.1)
Yes, our own protocols taken from the literature and arranged according to our own needs	120 (37.9)
No, I do not use a protocol	116 (36.6)
**What clinical results do you expect in patients who are getting postoperative pain support?**	
To reduce side effects as a result of insufficient postoperative pain control	204 (23.0)
Patient satisfaction	298 (33.6)
Surgeon satisfaction	30 (3.4)
Postoperative reduction in morbidity	183 (20.7)
Decreased frequency of nausea and vomiting	58 (6.5)
Shortening the discharge time	91 (10.3)
Reduction of cost	22 (2.5)
**What do you think should be done to improve postoperative pain treatment and apply it under appropriate conditions?**	
Creating a separate team for postoperative pain management in the hospital	209 (34.9)
To guide treatments by adhering to protocols	129 (21.5)
To train health care professionals for pain management	176 (29.4)
To inform patients in the preoperative period	85 (14.2)
**Do you think that postoperative pain treatment is effective and the treatment duration is enough in the institution where you work?**	
Yes	71 (22.7)
No	242 (77.3)
**If you think that the duration of the treatment is not enough in the institution where you work, what are the obstacle factors for postoperative pain treatment?**	
Lack of staff and time	212 (42.4)
Lack of equipment	84 (16.8)
Lack of medication	22 (4.4)
Excess workload	157 (31.4)
Insufficient patient information	25 (5.0)

%: column percentage, %^*^: calculated over the total answer given.

**Table 4.** Distribution of Approaches Toward Postoperative Pain Treatment According to Surgery

Variables	n (%)
What is your most preferred method for postoperative pain treatment for minor surgical interventions (herniography, gynecological laparoscopy, varicose veins, etc.)?*	
Paracetamol	141 (24.5)
Opioid	156 (27.1)
NSAID	178 (31.0)
Local anaesthetic infiltration of the wound site	39 (6.8)
Peripheral nerve blockade	26 (4.5)
Systemic opioid (PCA)	35 (6.1)
What is your most frequently preferred method for postoperative pain treatment for medium-sized surgical interventions (hip replacement, hysterectomy, maxillofacial surgery, etc.)?*	
Paracetamol	72 (11.7)
Opioid	143 (23.3)
NSAID	152 (24.8)
Local anaesthetic infiltration of the wound site	18 (2.9)
Peripheral nerve blockade	62 (10.1)
Systemic opioid (PCA)	166 (27.2)
What is your most preferred method for postoperative pain relief for major surgical interventions (thoracotomy, major abdominal surgery, knee surgery, etc.)?*	
Paracetamol	33 (5.5)
Opioid	109 (18.1)
NSAID	85 (14.1)
Local anaesthetic infiltration of the wound site	32 (5.3)
Peripheral nerve blockade	59 (9.8)
Systemic opioid (PCA)	132 (21.9)
Multimodal analgesia	152 (25.3)

%: Column percentage, %*: Calculated over the total answer given. PCA: patient-controlled analgesia, NSAID: non-steroidal anti-inflammatory drug.

**Table 5.** Distribution of Preferred Methods in Postoperative Pain Treatment

Variables	n (%)
**Which method do you use most frequently during postoperative pain treatment?**	
Intravenous patient controlled analgesia	157 (21.9)
Epidural patient controlled analgesia	137 (19.1)
Peripheral nerve blockade	52 (7.3)
Intermittent NSAID administration	186 (25.9)
Intramuscular/intravenous opioid as needed	177 (24.7)
Peripheral nerve blockade (PCA)	8 (1.1)
**When determining your method of choice, what are you emphasizing the most?**	
Physical state of the case	122 (14.0)
Severity of the pain	247 (28.4)
Severe pain expected time	71 (8.2)
Location and nature of the surgical intervention	229 (26.4)
Personnel and technical facilities	75 (8.6)
The risks of the method to the patient	125 (14.4)
**Which one do you prefer most frequently as a non-opioid in the postoperative multimodal approach?**	
Paracetamol	127 (36.5)
NSAID	186 (53.4)
Alpha-2 adrenergic agonist (clonidine, dexmedetomidine)	1 (0.3)
Gabapentin and pregabalin	1 (0.3)
Ketamine	1 (0.3)
Lidocaine infusion	3 (0.9)
Peripheral nerve blockade	13 (3.7)
Local anaesthetic infiltration	16 (4.6)
**Which opioids, that you can find in Turkey, do you prefer more frequently?**	
Hydromorphone (peroral)	4 (1.2)
Fentanyl (enteral)	59 (18.2)
Meperidine (enteral)	96 (29.6)
Morphine (enteral/peroral)	67 (20.6)
Codeine (peroral)	5 (1.5)
Tramadol (enteral/peroral)	94 (28.9)
**Which application method of pharmacological agents do you frequently prefer during postoperative pain treatment?**	
(Pro re nata) Application when necessary	44 (13.5)
Periodic application (intermittent)	136 (41.7)
Continuous infusion	15 (4.6)
PCA	131 (40.2)

%: column percentage, PCA: patient-controlled analgesia, NSAID: non-steroidal anti-inflammatory drug.

**Table 6.** Distribution of Preferred Methods in Postoperative Pain Treatment

Variables	n (%)
**Do you prefer preemptive analgesia for postoperative analgesia?**	
Yes	180 (57.3)
No	134 (42.7)
If the answer is no, what are the obstacles to preemptive analgesia preference?*	
Lack of scientific evidence	14 (7.0)
Difficulty of putting into practice	96 (48.1)
Adjusting the timing and duration of treatment	49 (24.8)
Failure to adjust the optimal dose and timing of drugs	20 (10.1)
Patient selection	16 (8.0)
Cost-effectiveness rate	4 (2.0)
**Do you use non-pharmacological methods for the treatment of postoperative pain?**	
Yes	19 (6.0)
No	296 (94.0)
If the answer is yes, which non-pharmacological methods do you prefer?*	
TENS	6 (25.0)
Electrode implantation	1 (4.2)
Acupuncture	4 (16.7)
Psychological premedication	10 (41.6)
Hypnosis	2 (8.3)
Biofeedback	-
Cryoanalgesia	1 (4.2)

%: column percentage, %*: calculated over the total answer given, TENS: transcutaneous electrical nerve stimulation
